# Synthesis, Electrocatalytic and Gas Transport Characteristics of Pentagonally Structured Star-Shaped Nanocrystallites of Pd-Ag

**DOI:** 10.3390/nano10102081

**Published:** 2020-10-21

**Authors:** Iliya Petriev, Polina Pushankina, Ivan Lutsenko, Nikita Shostak, Mikhail Baryshev

**Affiliations:** 1 Department of Physics, Kuban State University, 350040 Krasnodar, Russia; polina_pushankina@mail.ru (P.P.); vanke08@mail.ru (I.L.); baryshev_mg@mail.ru (M.B.); 2 Laboratory of Problems of Stable Isotope Spreading in Living Systems, Southern Scientific Centre of the RAS, 344000 Rostov-on-Don, Russia; 3 Department of Oil and Gas Business, Kuban State Technological University, 350040 Krasnodar, Russia; shostak@kubstu.ru

**Keywords:** nanostars, nanostructured surface, palladium-containing membranes, catalytic activity, methanol oxidation, hydrogen permeability

## Abstract

The method of synthesis of bimetallic Pd–Ag pentagonally structured catalyst “nanostar” on the surface of Pd-23%Ag alloy films has been developed. The resulting catalyst was studied as a highly active functional layer for methanol oxidation reaction (MOR) in alkaline media and the intensification of hydrogen transport through the Pd-23%Ag membrane in the processes of hydrogen diffusion purification. A modifying layer with a controlled size, composition and excellent electrocatalytic activity was synthesized by electrochemical deposition at a reduced current density compared to classical methods. The low deposition rate affects the formation of pentagonally structured nanocrystallites, allowing Pd and Ag particles to form well-defined structures due to the properties of the surfactant used. Electrochemical studies have demonstrated that the catalyst synthesized by the “nanostar” method shows better electrocatalytic activity in relation to MOR and demonstrates a higher peak current (up to 17.82 µA cm^−2^) in comparison with one for the catalyst synthesized by the “nanoparticle” method (up to 10.66 µA cm^−2^) in a cyclic voltammetric study. The nanostar catalyst electrode releases the highest current density (0.25 µA cm^−2^) for MOR and demonstrates higher catalytic activity for the oxidation of possible intermediates such as sodium formate in MOR. In the processes of diffusion membrane purification of hydrogen, a multiple increase in the density of the penetrating flux of hydrogen through the membranes modified by the “nanostar” catalyst (up to 10.6 mmol s^−1^ m^−2^) was demonstrated in comparison with the membranes modified by the “nanoparticles” method (up to 4.49 mmol s^−1^ m^−2^). Research data may indicate that the properties of the developed pentagonally structured catalyst “nanostar” and its enhanced activity with respect to reactions involving hydrogen increase the desorption activity of the membrane, which ultimately accelerates the overall stepwise transfer of hydrogen across the membrane.

## 1. Introduction

According to the latest research in the field of nanomaterials [1,2,3,4,5,6,7,8], nanoparticles of noble metals are already widely used in almost all spheres of human life and activity. Of particular interest is the potential of their application in the field of alternative energy, which is conditioned by the unusual catalytic, chemosorption, magnetic and other properties of nanoparticles.

Electrocatalysis is one of the areas of usage of noble metal nanoparticles in the field of resource-saving energy. Platinum group metals are considered universal catalysts in alcohol oxidation and oxygen reduction reactions (ORR) [9,10,11], which makes their usage preferable in direct alcohol fuel cells. In this application, palladium is the most promising replacement for platinum as an electrocatalyst in alkaline media with improved anodic kinetics of methanol oxidation and higher resistance to CO poisoning [12,13,14]. In recent years, a class of bimetallic nanoparticles became the most preferable which is conditioned by a strong synergistic effect with increased catalytic activity and durability, compared to monometallic analogues [15]. In this way, the most promising approach is to regulate the reactivity of palladium by doping it with silver [16,17,18,19,20]. Based on the theory of d-bands [21] and the calculations of Hammer and Nørskov [22], the center of the d-zone of palladium with a lattice value of 3.89 Å shifts up when combined with silver with a lattice value of 4.09 Å [23], which leads to greater adsorption of the OH– ion and, consequently, to an increase in the rate of oxidation of alcohols [17].

In addition, highly-dispersed nanostructured bimetallic particles based on noble metals are of interest in membrane applications, where noble metal nanoparticles can be used as functional surface modifiers [24,25,26,27]. This application makes it possible to accelerate the surface stages of hydrogen transport and thus intensify the process of hydrogen transport through metal membranes that are in the SLR mode (surface limited regime). The disadvantage of monolithic metal membranes is their relatively low permeability at low temperatures (<100 °C). This disadvantage can be eliminated by modifying the surface of the metal film with a highly developed coating, by applying metal nanoparticles Pt, Pd, Fe, Co, Ni and Cu to the input and output surfaces of all-metal membranes [28,29] or by calcination in air [30,31]. Highly dispersed modifiers increase the actual working surface, which leads to an increase in the number of chemisorption centers, the role of which is most often performed by the corners and faces of crystallites. In this case, most of the octohedral internodes will belong to the surface [32], which facilitates the transport of hydrogen and reduces the probability of capturing the hydrogen atom by various kinds of defects. Therefore, a highly dispersed hydrogen-absorbing surface layer in contact with compact samples of metal films can significantly accelerate the dissolution of hydrogen at room temperature, or make this dissolution even possible.

In many applications and processes, as in the ones described above, the activity of bimetallic nanocrystallites strongly depends on their size, active surface area and morphology [33,34]. Therefore, in recent years, a large number of noble metal-based catalysts with various morphologies have been synthesized, such as monodisperse nanoparticles [35], nanowires [36], nanostars [37,38,39], nanopores [40], nanoplast arrays [41], nanostructures [42], nanoflowers [43,44], etc. Special attention is being paid to structures with five-fold symmetry, which is considered forbidden in classical crystallography, but is acceptable for ultra-disperse conditions with the size of metal particles in the range from 1 to 100 nm [45]. A distinctive feature of Pd particles is their high crystallinity and the possibility of forming a fivefold symmetry, which is confirmed by a lot of studies [46,47,48,49,50,51]. These structural units, called clusters, are crystalline fragments, usually with significant distortions, and non-crystalline, mostly with fivefold symmetry. Nanoclusters based on noble metals, which are symmetrical structures, are characterized by the phenomenon of multiple doubling [52]. Three-dimensional atomic configurations of such crystallites [53] have a narrow size distribution and small cluster forms containing from a few units to several hundred atoms, the majority of which are concentrated on the surface. These structures are characterized by a correct geometric shape and the formation of energy-preferred one or more axes of the fifth order. The main models describing the growth mechanism of pentagonal structures are the Mackay icosahedron [54,55], the Ino decahedron [56] and the truncated Marx decahedron [57]. According to the theories of these models, polyedric nanocrystallites are characterized by the accumulation of a large proportion of surface atoms.

Nanostructures called “nanostars” are of particular interest. Numerous studies of such morphologies have been conducted for gold or bimetals based on it [58,59,60]. Such structures can resemble “thorns” in shape [38,60], and also have a fivefold symmetry, reproducing the shape of regular five-pointed stars [58,61]. Presumably, the lateral growth of pentagonal bipyramids along the borders of the twins contributes to the formation of pentabranched particles. Such pentagonally structured surface modifiers with enhanced electrocatalytic activity in the methanol oxidation reaction can find their application in direct methanol fuel cells. In addition, the materials being developed, due to their ability to significantly accelerate the surface stages of hydrogen transport (dissociative adsorption and recombinant desorption) through all-metal membranes at low temperatures (up to 100 °C), can find their application in the processes of membrane diffusion production of ultrapure hydrogen.

In general, the search for and discovery of new nanostructures is incredibly important today because it can have useful applications in various spheres of life due to the importance of nanotechnology in current and future applications. Based on the foregoing, the aim of the article was to synthesize pentagonally structured bimetallic Pd–Ag catalysts of a new morphology and investigate their properties and characteristics in two practical applications: methanol oxidation and membrane hydrogen evolution. The applied part of the research is also aimed at implementing and improving the most promising methods for modifying hydrogen-permeable palladium-based membranes to achieve the highest values of hydrogen flux at low temperatures up to 100 °C.

## 2. Materials and Methods

### 2.1. Synthesis of Bimetallic Pd–Ag Nanoparticles

**Figure 1:**
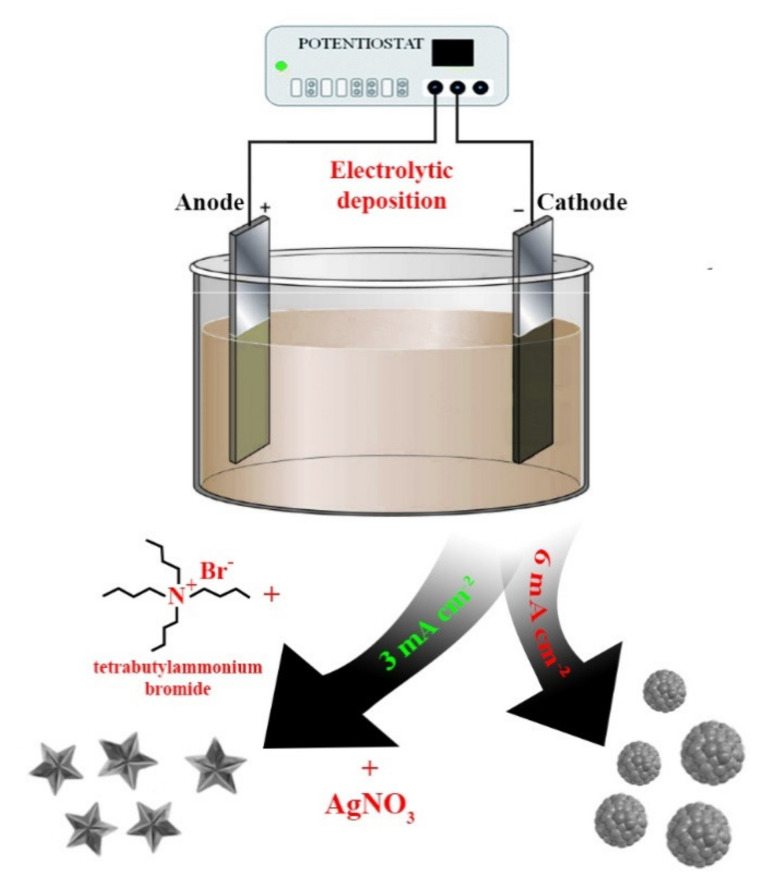
Schematic diagram of two methods for the synthesis of bimetallic Pd–Ag nanoparticles: “nanostar” and “nanoparticle”.

Two methods were used for the synthesis of Pd–Ag bimetallic particles on the surface of Pd–23%Ag alloy films (Figure 1) with a thickness of 10 μm, purchased from the LLC Research and Production company “Specialized metallurgy”, Yekaterinburg, Russia:

1. The classic palladium-black method or the “nanoparticle” method. The Pd–23%Ag alloy film was fixed in a holder, washed in 96% ethanol (Vecton, Saint-Petersburg, Russia), degreased by boiling for 30 min in a concentrated 6 M NaOH (Vecton, Saint-Petersburg, Russia) solution, then transferred for etching to a 60% HNO_3_ (Vecton, Saint-Petersburg, Russia) solution for 30 s, after which it was immediately transferred to a vessel with running distilled water for 10 min. Then, the film on an inert holder was transferred to an electrolytic cell for coating. The mass of the holder made of silver (LLC Research and Production company “Specialized metallurgy”, Yekaterinburg, Russia) with a purity of 99.99% was used as the current supply of the cathode. The contact was made with silver wire. Then, the palladium–silver alloy film was transferred to a cell with 0.1 M HCl (Vecton, Saint-Petersburg, Russia)and anodically polarized at a current density of 10–20 mA cm^−2^ using a potentiostat–galvanostat P-40X (Electrochemical Instruments, Chernogolovka, Russia), washed, cathodically polarized in 0.05 M H_2_SO_4_ (Vecton, Saint-Petersburg, Russia) at a current density of 10–20 mA cm^−2^, then filled with a 2% solution of H_2_PdCl_4_ (Vecton, Saint-Petersburg, Russia). Palladium black deposition was performed at a current density of 5–6 mA cm^−2^ for 30 min, after which it was washed with bidistillate and cathodically polarized in 0.05 M H_2_SO_4_ (Vecton, Saint-Petersburg, Russia).
2. The difference of the “nanostar” method from the previous one is that, after washing with bidistillate, the cell was filled with a solution containing, along with H_2_PdCl_4_ (2%; Vecton, Saint-Petersburg, Russia), tetrabutylammonium bromide (0.01 mol L^−1^; Vecton, Saint-Petersburg, Russia) as a surfactant and AgNO_3_ (0.005 mol L^−1^; Vecton, Saint-Petersburg, Russia). Deposition was performed at a reduced current density, compared to the previous method, 3–4 mA cm^−2^ in stages with a step of 5 min for 30 min. In other words, in order to control changes in surface morphology during electrolytic deposition, a sample was selected after each stage for electron microscopy by cutting off part of the film. For this reason, the current density was adjusted over the area of the electrode for the further process.

All experimental samples of Pd-23%Ag films were modified on both sides.

The chemical composition of the obtained alloys was controlled by microrentgenospectral analysis on an INCA (Oxford) semiconductor energy dispersion attachment part of a JEOL JSM-7500F scanning electron microscope (JEOL, Tokyo, Japan).

Electron microscopy was performed in SE (secondary electron) mode using a JEOL JSM-7500F scanning electron microscope (JEOL, Tokyo, Japan). Microscopy data processing and statistical parameters calculation were performed using the modular data visualization and analysis program Gwyddion.

### 2.2. Electrochemical Measurements

Electrocatalytic reactions of methanol oxidation were studied using a cyclic voltammetric (CV) method at room temperature (25 °C) using a three-electrode cell on an automated potentiostat–galvanostat Elins P-40X (Electrochemical Instruments, Chernogolovka, Russia). Pd-23%Ag electrodes modified with different types of coatings were used as working electrodes. Reference electrode: Ag/AgCl electrode (Electrochemical Instruments, Chernogolovka, Russia). All the measurements presented used a platinum counter electrode; palladium foil was used as a counter electrode in some measurements. CV multiscan was performed in the operating potential range from −0.9 V to +0.5 V with a scanning speed of 50 mV s^−1^ in 1.0 M water solution of NaOH (Vecton, Saint-Petersburg, Russia) with 0.5 M methanol (Vecton, Saint-Petersburg, Russia). The currents were normalized to the geometric area of the electrodes, and all potentials are reported relative to the silver chloride electrode.

Chronoamperometric (CA) studies were conducted for 2400 s at a constant potential of −0.3 V to investigate the relative stability of the electrodes.

### 2.3. Measurement of Hydrogen Permeability

**Figure 2:**
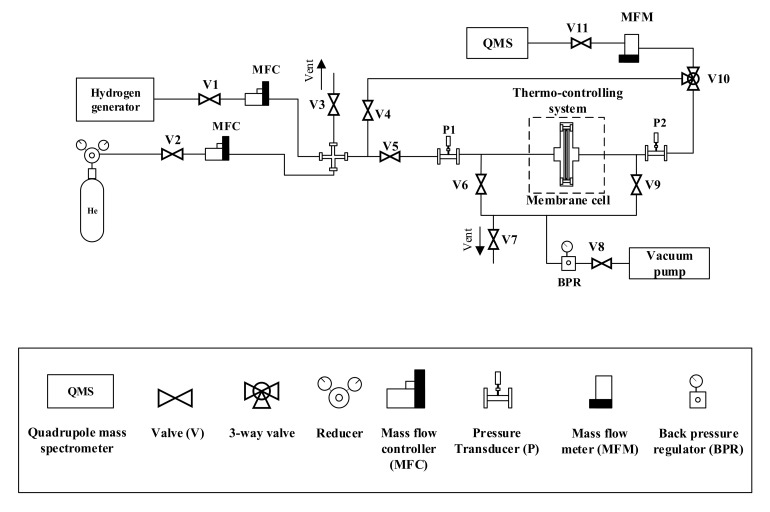
Diagram of the installation for measuring hydrogen permeability.

The scheme of the experimental set up for measuring hydrogen permeability is shown in Figure 2.

Before the experiment, helium (99.999%; LLC Hydrogen technologies, Krasnodar, Russia) was fed into the system for purging and leak testing. Hydrogen with a purity of 99.999% (obtained from hydrogen generator Spektr, Nizhniy Novgorod, Russia) supplied to the input side of the membrane at a pressure in the range of 0 to 0.3 MPa at a temperature in the range of 25 to 100 °C. The supply of both gases to the system was controlled by mass flow controller that provided the required flow rate. The permeability was measured using a diffusion cell that provides reliable fixation and sealing of the membrane sample. The membrane with a working area of 0.95 cm^2^ was mounted in the cell using argon-arc welding and additionally fixed with a flange connection.

Measurement of the penetrating flux has produced in the vacuum created in the behind-membrane part of the system. The vacuum level as well as the pressure on the input side of the membrane were monitored using pressure transducers. The analysis of the diffused hydrogen was performed using a quadrupole mass spectrometer.

The experimental method used aimed at establishing the fundamental laws of hydrogen transport through metal membranes at a low temperature (from 25 to 100 °C).

## 3. Results and Discussion

### 3.1. Morphology and Characteristics of Synthesized Nanoparticles

During the research original and reproducible methods for creating bimetallic palladium–silver catalysts on the surface of Pd-23%Ag films with controlled morphology were developed. Two series of samples were produced: in the first series, the catalyst was synthesized by the “nanoparticle” method; in the second series, the “nanostar” synthesis method was used.

**Figure 3:**
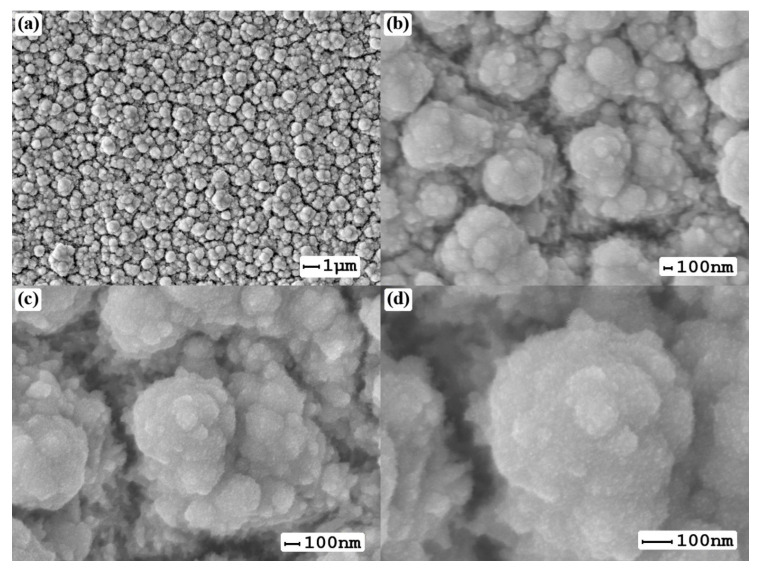
SEM images of the surface of palladium–silver films with a modified surface using the “nanoparticle” method at magnifications of 5000 (**a**), 30,000 (**b**), 50,000 (**c**) and 100,000 (**d**).

**Figure 4:**
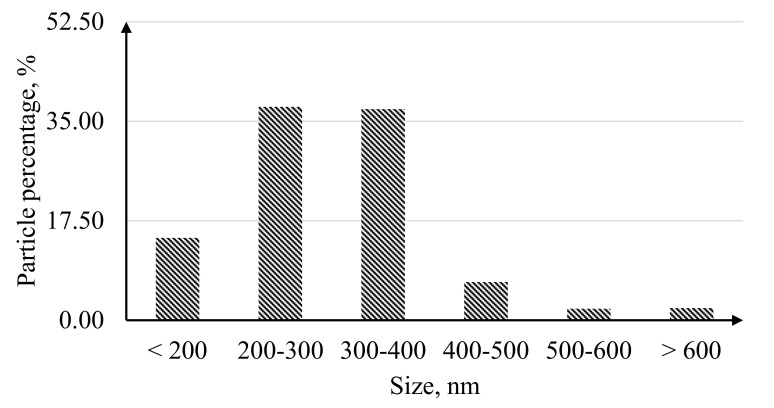
Histogram of particle size distribution obtained by the “nanoparticle” method.

Microphotographs of the first series of catalysts synthesized using the “nanoparticle” method are shown in Figure 3. The histogram of the particle size distribution demonstrated in Figure 4 shows 75% of the particles obtained by this method have a size range of 200–400 nm.

**Figure 5:**
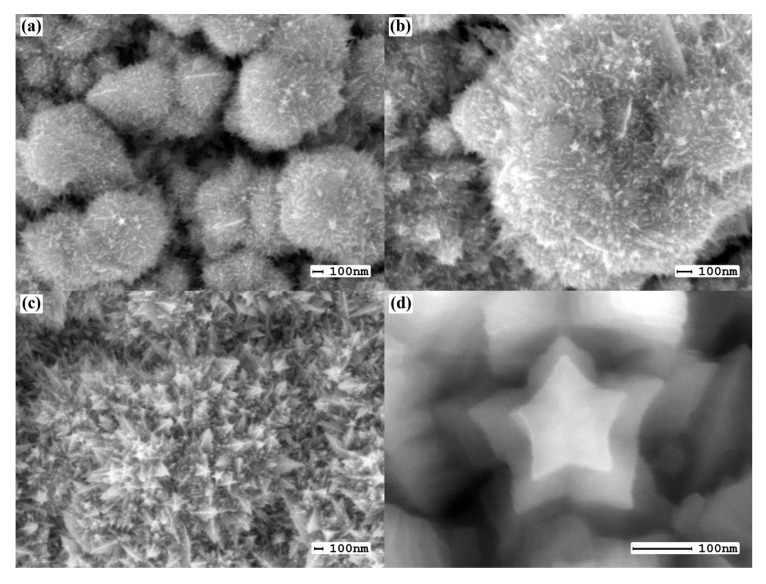
SEM images of the surface of Pd–Ag membranes modified by the “nanostar” method after electrolytic deposition for 5 min (**a**), 15 min (**b**) and 25 min (**c**,**d**).

Microphotographs of the surfaces of the second series of samples modified by the “nanostar” method are shown in Figure 5. This name was chosen due to the fact that the nanocrystallites of the formed coating resembled stars in shape. The study of such particles is given in prior research [37].

**Figure 6:**
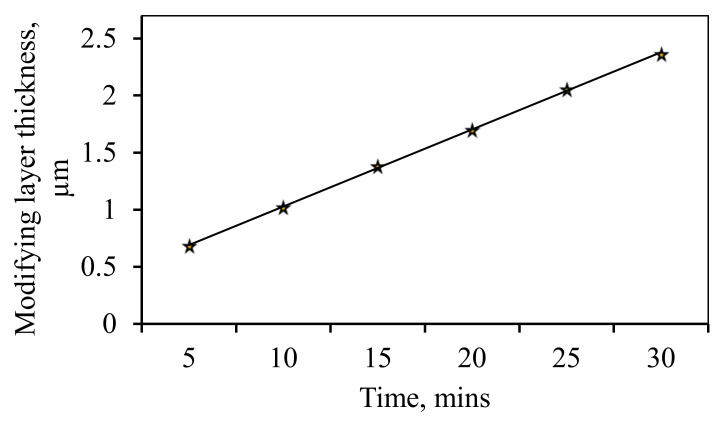
Graph of the dependence of the thickness of the modifying coating of the “nanostar” type on the time of film deposition.

**Figure 7:**
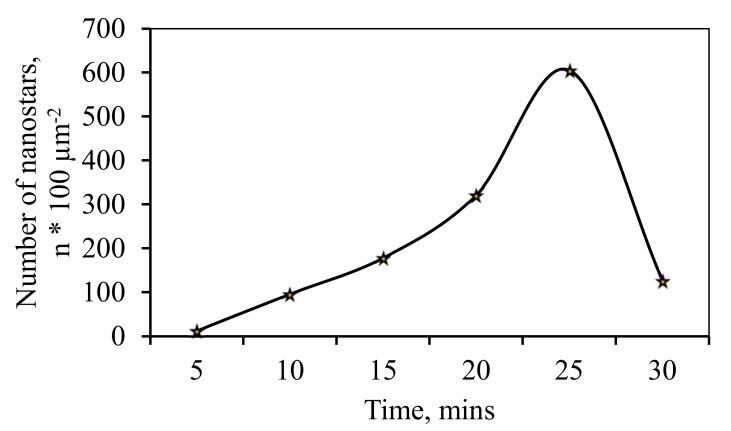
Graph of the dependence of the number of “nanostars” on the area of 100 µm^2^ on the time of film deposition.

In the electrolytic deposition of the coating it was found that with increasing duration of deposition time, there was growing not only the thickness of the modifying layer of the coating (Figure 6), but increase in the number of “nanostar” on the surface was observed (Figure 7). Stable increase in the number of separately taken star nanocrystallites recorded up to 25 min, followed by a sharp decline. The maximum number of “nanostar” was recorded at 25 min and was 605.4 per 100 µm^2^.

**Figure 8:**
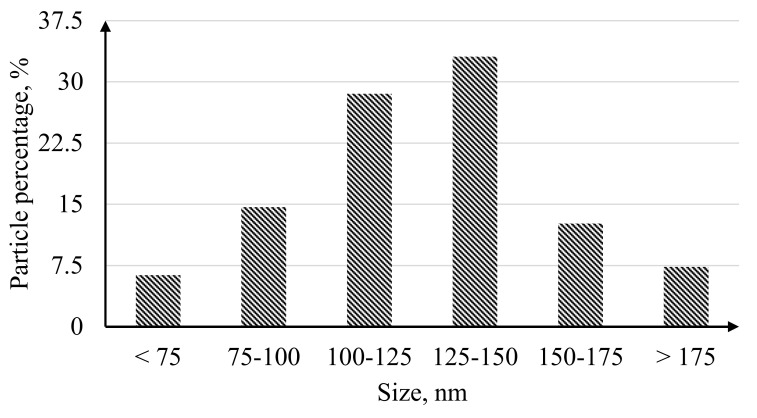
Histogram of the size distribution of nanoparticles obtained by the “nanostar” method.

The histogram of the nanoparticle size distribution shown in Figure 8 demonstrated that 62% of the catalyst particles synthesized on the surface of the Pd-23%Ag film by the “nanostar” method were in the size range of 100–150 nm.

Most of the star-shaped nanoparticles obtained in other research were synthesized in colloidal solutions. In our case, pentagonally-branched palladium crystallites are obtained on the substrate in the form of Pd-23%Ag film, which is a significant difference from the structural configurations obtained by other authors. The role of the stabilizer is performed by a metal palladium–silver foil, which is both the base of the electrode and the membrane. This can help prevent unwanted agglomeration and growth, as in works [62,63]. In addition, the role of a stabilizer is performed by a ligand that is part of the surfactant tetrabutylammonium bromide, the structure of which probably contributes to the formation of deposited particles in this form.

**Figure 9:**
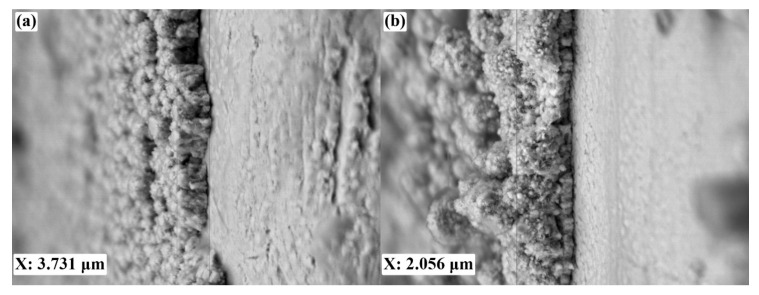
SEM images of sections of palladium–silver films modified by “nanoparticles” (**a**) and “nanostar” (**b**) methods.

The reduced current density, compared to the “nanoparticle” method, is a factor that allows a maintaining of the initially set character of the growth of structures from atomic to micron sizes of crystallites. The reduced current density allows the particles to occupy energy-efficient positions for a short time during deposition and makes it possible to create a coating of a smaller thickness. This leads to a significant reduction of the amount of noble metal used to create an electrochemically modified film. Therefore, the average thickness of the catalyst layer for samples modified by the “nanoparticle” method was 3.731 microns (Figure 9a); modified by the “nanostar” method, 2.056 microns (Figure 9b).

**Figure 10:**
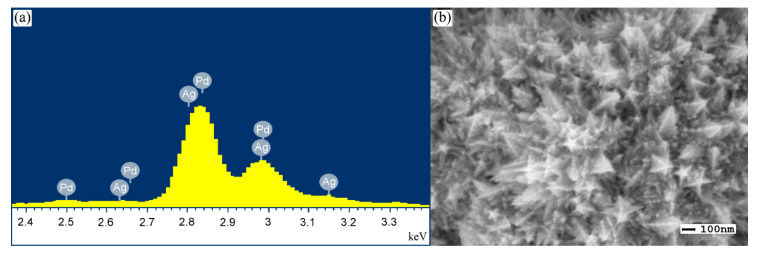
SEM image (**a**) and EDX spectra of Pd–Ag membrane samples modified by “nanostar” method (**b**).

X-ray microanalysis was performed on an Inca (Oxford) JEOL JSM-7500F semiconductor energy dispersive attachment to determine the elemental composition of the modifying coating. Analysis of the pentagonally branched catalyst synthesized by the “nanostar” method showed (Figure 10) that the atomic percentage of palladium and silver elements in the functional layer was 88.32% Pd, 11.68% Ag.

Table 1. Areas of samples with equal projected surface areas of 12 µm2 were studied. The obtained data shows that when modifying the surface of Pd-23%Ag foil by the “nanoparticle” method, the actual working surface area of the sample increased and the roughness coefficient was 12.3. The functional layer deposited by the “nanostar” method on the surface of the Pd-23%Ag film demonstrated a higher roughness coefficient of 20.5.

The researches carried out allow us to make assumptions about the mechanism of growth of atypical for classical crystallography structures of catalyst particles synthesized by the “nanostar” method. In the process of electrolytic deposition, silver atoms, falling on the substrate in a certain way, form geometrically correct bipyramides together with palladium atoms. Further, by repeated doubling of the obtained particles, faceted nuclei are formed, which are decahedra with a single axis of the fifth order. Each of the five double borders along the sides of the decahedron forms a branch formed by lateral growth. The formed nanocrystallite looks like a regular five-pointed star with symmetrical branches. This kind of symmetry is considered forbidden in classical crystallography, but it is acceptable for ultrafine media and can be observed in quasicrystals with non-periodic arrangement of atoms.

### 3.2. Cyclic Voltammetric Study of Methanol Oxidation in Alkaline

**Figure 11:**
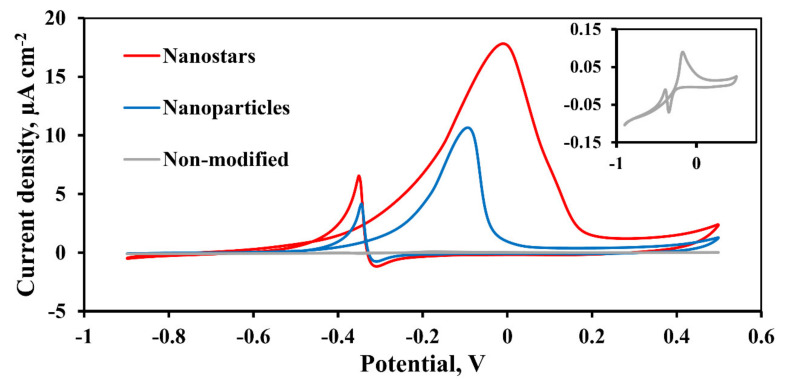
The cyclic voltammetric (CV) profile of steady cycles (30th) presented in current density in µA cm^−2^ of the Pd-23%Ag electrode without a catalyst and of the Pd-23%Ag electrodes with “nanoparticle” and “nanostar” type catalysts for alkaline methanol oxidation in 0.5 M methanol in 1.0 M NaOH at room temperature with a scanning potential rate of 50 mV s^−1^.

The CV of a smooth non-modified Pd-23%Ag electrode (inset in Figure 11) and developed electrodes based on Pd-23%Ag film modified by the “nanoparticle” and “nanostar” (Figure 11) methods, durable to the methanol oxidation reaction (MOR), were taken at a scanning speed of 50 mV s^−1^ in the potential range from −0.9 to +0.5 V. This range was chosen because of the deep evolution of hydrogen and oxygen occurs when the potential is below the negative and above the positive threshold values of the potential, respectively. During the anode scan, a large distinct peak of methanol oxidation can be observed for Pd-23%Ag electrodes modified with nanoparticle and “nanostar” catalysts at 10.66 µA cm^−2^ and 17.82 µA cm^−2^. The negative peak potential bias for both developed electrodes indicates that they are good catalysts in the methanol oxidation reaction. The occurrence of oxidation peaks during reverse (cathodic) scanning at 6.55 µA cm^−2^ for the nanoparticle catalyst and at 4.13 µA cm^−2^ for the “nanostar” catalyst is associated with the removal of chemisorpted CO, and also with the iterating of methanol oxidation by fresh adsorption [48].

Area-normalized peak current densities on the anode (i_F_) and cathode (i_B_) sweeps (Table 2) show that the Pd-23%Ag electrode modified by the “nanostar” method is the best among the studied electrodes. Large values of i_F_ and i_B_ for electrodes modified by the “nanostar” method may be due to a larger electrochemical area. It also can be noted that the increased i_B_ values of the developed electrodes, compared to the smooth palladium–silver electrode, which means greater formate adsorption, leading to blocking of the electrode surface at a higher potential and removal at a lower potential. It should also be noted that the combination of palladium and silver can demonstrate enhanced electrocatalytic activity of the developed catalysts in relation to the methanol oxidation reaction in an alkaline media and most effectively inhibit the poisoning of palladium active sites, which can be confirm in [15,17]. This effect is explained by the ability of silver to accelerate the oxidation of reaction intermediates, since Ag_2_O and AgOH serve as storage materials of Pd(OH).

The i_F_/i_B_ ratio is used to evaluate the resistance of a catalyst to CO poisoning [64]. Low i_F_/i_B_ values usually indicate poor methanol oxidation to CO_2_ and excessive accumulation of residual forms of carbon on the catalyst surface, while higher i_F_/i_B_ values indicate more efficient CO desorption on the catalyst under study. For a non-modified electrode, the i_F_/i_B_ ratio showed a fairly low value of only 0.23, which may indicate a low efficiency of removal of toxic carbon-containing substances. The i_F_/i_B_ values obtained for the developed catalysts of the “nanoparticle” and “nanostar” type were 2.58 and 2.72, respectively, which indicates a distinctly increased efficiency of removing CO poisonous particles from the catalyst surface.

**Figure 12:**
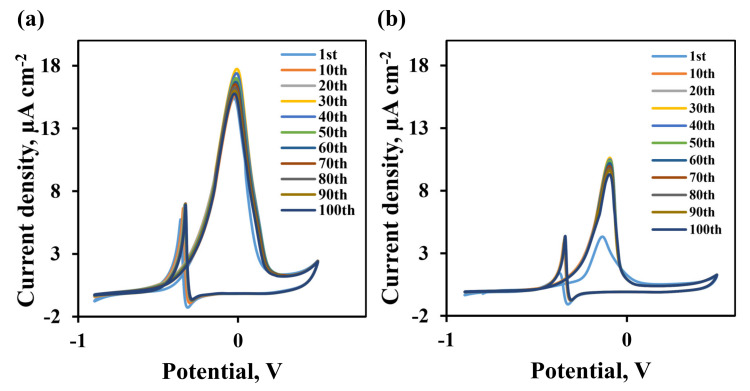
Multiscanning of Pd-23%Ag CV electrodes with “nanostar” (**a**) and “nanoparticle” (**b**) catalysts up to 100th cycle in 0.5 M methanol in 1 M NaOH at 50 mV s^−1^ scan rate.

The stability of the developed electrodes modified by the “nanoparticle” and “nanostar” methods was studied by multi-scanning of 100 CV cycles. From the first cycle, a direct peak is registered due to the oxidation of methanol on the Pd surface. From voltamperograms shown in Figure 11 it is demonstrated that the current density of both electrodes under study increased and was established by the 30th cycle. After the 30th cycle, the maximum peak current density is reached, which is 17.82 µA cm^−2^ (Figure 12a) for an electrode with a pentagonally branched “nanostar” catalyst and 10.66 µA cm^−2^ for an electrode with a “nanoparticle” catalyst (Figure 12b). The peak current density decreases for an electrode with a “nanostar” catalyst by only 11.55% per 100 cycle, and for an electrode with a “nanoparticle” catalyst, by 12.3%. This makes it clear that the electrodes we have developed demonstrate significant stability over several cycles.

### 3.3. Chronoamperometric Study

**Figure 13:**
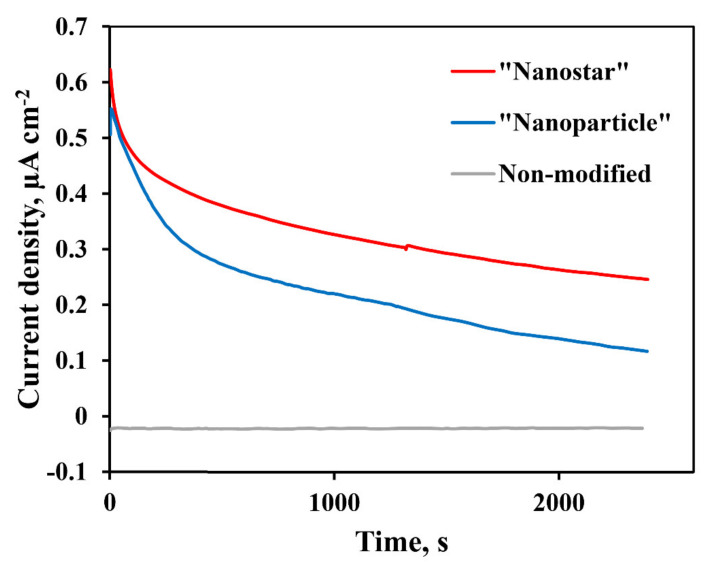
Chronoamperometric profiles for Pd-23%Ag electrodes without catalysts and for Pd-23%Ag electrodes with “nanoparticle” and “nanostar” catalysts for 0.5 M methanol in 1 M aqueous NaOH solution at potential of −0.3 V up to 2400 s.

The CA studies were performed to further evaluate the activity and long-term stability in methanol oxidation reactions for a smooth non-modified Pd-23%Ag electrode and developed Pd-23%Ag electrodes modified by the “nanoparticle” and “nanostar” methods. The measurements were performed at a constant potential of −0.3 V in a solution of 0.5 M methanol in 1 M NaOH for 2400 s. Chronoamperometric profiles are shown in Figure 13. Both catalysts showed fairly similar characteristics in the methanol electrooxidation reaction. High initial current density was determined by the double layer charging and numerous active centers on the surface of the synthesized catalysts. Further, a gradual decrease of the current was recorded, which implies the formation of intermediate products, such as CO, and their accumulation on the active surface sites. Over time, a pseudo-stabilized state was observed with an almost constant current density for all electrodes. The pentagonally structured catalyst synthesized by the “nanostar” method demonstrated a maximum current density of 0.25 µA cm^−2^, which indicates its superiority over the catalyst synthesized by the “nanoparticle” method at –0.12 µA cm^−2^.

### 3.4. Study of Hydrogen Permeability

The positive effect of our coatings can be achieved only if the processes occurring on the surface of the membrane are limiting and the diffusion rate does not affect the total flux of hydrogen. As is known, this is possible for H_2_ adsorption on the feed side of the membrane only at high temperatures, when the hydrogen adhesion coefficient is low, or at low temperatures, when H_2_ desorption on the permeate side of the membrane affects the rate of hydrogen penetration. Such a guaranteed temperature for palladium membranes of various thicknesses, up to 100 microns, will be a temperature of 100 °C [65]. Therefore, the surface modifier can have a positive impact on the speed of flux of surface processes below 100 °C. In this case, the limiting stage is most likely desorption. Adsorption will only be important at very low partial pressures of hydrogen, when the hydrogen adhesion coefficient is low, or when there is significant surface contamination. Although most existing data on permeability for thick palladium membranes correspond to calculations for diffusion-limited permeability [66,67,68,69,70,71,72], there are significant discrepancies for membranes less than 10 microns thick [30,73,74,75,76,77,78]. Therefore, the range up to 100 °C was chosen as the most interesting, since it is in this range that the presence of a modifying coating plays a key role in the process of accelerating the flux of hydrogen through the membranes.

**Figure 14:**
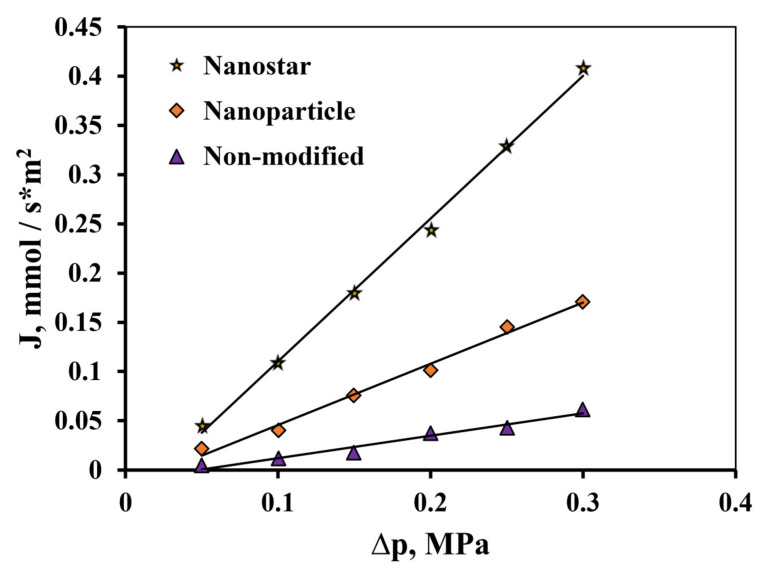
Dependence of the flux on the excess pressure of hydrogen at 25 °C on the input side of the unmodified Pd-23%Ag membrane and Pd-23%Ag membranes modified by the “nanoparticle” and “nanostar” methods.

**Figure 15:**
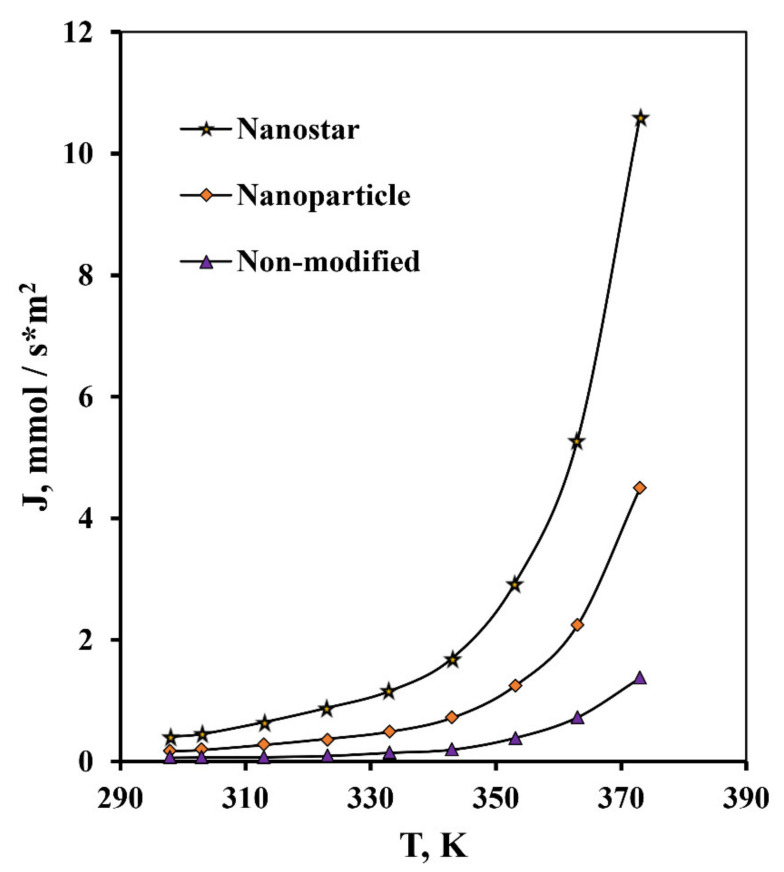
Temperature dependence of the hydrogen flux at ∆p = 0.3 MPa through a non-modified Pd-23%Ag membrane and Pd-23%Ag membranes modified by the “nanoparticle” and “nanostar” methods.

In studies of the hydrogen transport process, the highest values of the hydrogen flux in the high-pressure area up to 0.3 MPa (Figure 14) have been demonstrated by Pd-23%Ag membranes modified by the “nanostar” method up to 10.6 mmol s^−1^ m^−2^, which significantly exceeds the value of this indicator for membranes modified by the classical palladium black method, up to 4.49 mmol s^−1^ m^−2^. Membranes with a pentagonally structured functional layer also showed the highest values of flux up to 1.37 mmol s^−1^ m^−2^ with an increase in temperature from 25 °C to 100 °C (Figure 15). These values were 2.4 times higher than for the membranes modified by the “nanoparticle” method, and exceeded the values for the non-modified Pd-23%Ag membrane by a record 7.7 times.

The data obtained may indicate that the properties of the developed pentagonally structured “nanostar” catalyst and its enhanced activity with respect to reactions involving hydrogen increase the desorption activity of the membrane, which ultimately accelerates the overall stepwise transfer of hydrogen across the membrane, as shown previously [79]. In our opinion, this is the reason for the decrease in the energy barrier of the desorption–recombination process of hydrogen molecules on the membrane surface, which leads to an increase in the rate of hydrogen penetration through the membrane based on the Pd-23%Ag alloy. A significant increase in hydrogen permeability through nanostar-coated membranes, in comparison with a less pronounced increase in surface roughness, may indicate that the increase in desorption activity of the membrane surface is due not only to an extensive path—increasing the development and specific area of the coating—but also to an intensive one, creating a given structural organization of the modifying coating.

## 4. Conclusions

In this study, a new approach to the synthesis of a highly active bimetallic Pd–Ag catalyst with a completely new surface morphology never achieved by other methods has been demonstrated. The catalyst is nanocrystallites with non-classical for crystal physics fifth-order symmetry, visually resembling a star in shape. It was found experimentally that the formation of a pentagonally structured functional layer on the surface of a Pd-23%Ag film is possible only at reduced current densities (3–4 mA cm^−2^) in comparison with classical methods. The low deposition rate of Pd and Ag particles on a thin palladium–silver film allows them to form well-defined structures, probably due to the properties of the surfactant used—tetrabutylammonium bromide.

Studies of catalysts synthesized on the surface of a Pd-23%Ag film in the reaction of methanol oxidation in an alkaline media have revealed an improved and stable catalysis of a pentagonally structured catalyst compared to the catalyst obtained by the classical palladium black method. The electrode demonstrated a high current density (up to 17.82 µA cm^−2^) for MOR and higher catalytic activity for the oxidation of possible intermediates such as sodium formate. It is also noted that the combination of Pd and Ag is an effective method for improving the catalytic properties of the catalyst and its resistance to poisoning.

Using the developed catalysts, the surface of Pd-23%Ag membranes was modified and their effect on the kinetic characteristics of the hydrogen transfer process was investigated. A multiple increase in the density of the penetrating flux of hydrogen through the membranes modified by the “nanostar” method compared to the membranes modified by the “nanoparticle” method has been demonstrated. The difference in penetrating fluxes reaches a value of 6.11 mmol s^−1^ m^−2^, which is an increase of 2.4 times. Under low temperature conditions, the obtained values are quite significant, since there is practically no stable detectable permeability in this temperature range. The results obtained have shown that the developed methods can significantly increase the productivity of Pd–Ag membranes (increase the hydrogen flux) in the low-temperature range up to 100 °C. It is very likely that this will also allow to increase the palladium membranes durability, since hydrogen embrittlement will be partially eliminated due to the absence of thermal cycling phases in a hydrogen atmosphere. Therefore, in the applied part of the study, the task was to obtain palladium-containing films capable of hydrogen permeation at a high rate precisely at low temperatures. From a practical point of view, this task is the key one, since its solution makes it possible to create low-temperature gas-diffusion hydrogen electrodes and oxygen–hydrogen fuel cells based on them. From a theoretical point of view, this problem also seems to be extremely urgent, since it gives an idea of the mechanisms of hydrogen transfer by metals. Therefore, the achievement of the highest values of the hydrogen flux through metal membranes at low temperatures can be of both theoretical and practical interest in this area.

All the data obtained in the study of the hydrogen permeability and catalytic activity of the developed Pd-23%Ag films modified by the “nanoparticle” and “nanostar” method are closely correlated with each other, which is proof of the correctness of the results. They also confirm the effectiveness and versatility of the developed catalyst of the new nanostar morphology for various applications in current and future applications in the field of nanotechnology.

## Figures and Tables

**Table 1:** Statistical data of surface morphology parameters of films modified by two different methods.

Type of Modifying Coating	Palladium Black (Nanoparticles)	Nanostars
Average value, µm	1.13074	1.00247
RMS roughness (Sq), nm	223.578	315.565
Mean roughness (Sa), nm	181.259	256.207
Coefficient of roughness	12.27	20.53
Skew (Ssk)	−0.334003	0.27527
Excess kurtosis	−0.384457	−0.34419
Projected area, µm^2^	12	12
Surface area, µm^2^	147.25	246.4
Volume, µm^3^	13.57	12.0297
Variation, µm^2^	164.14	269.66
Inclination θ, deg	1.53	1.73
Inclination φ, deg	20.50	143.9

**Table 2:** Statistical parameters obtained from cyclic voltammetric (CV) studies of the studied electrodes immersed in 0.5 M methanol in 1 M NaOH solution at room temperature.

Electrodes	R_f_ (Coefficient of Roughness)	E_F_, V	i_F_, µA cm^−2^	E_B_, V	i_B_, µA cm^−2^	i_F_/i_B_
Non-modified	1.56	−0.17	0.09	−0.01	−0.39	0.23
Nanoparticles	12.27	−0.09	10.66	−0.34	4.13	2.58
Nanostars	20.53	−0.01	17.82	−0.35	6.55	2.72
